# Weak Point of SARS-CoV-2: Human and Viral Ion Channels under External Physical Fields

**DOI:** 10.3390/ijms232315185

**Published:** 2022-12-02

**Authors:** Andrzej Fuliński

**Affiliations:** Institute of Theoretical Physics, Jagiellonian University, Łojasiewicza 11, 30-348 Kraków, Poland; andrzej.fulinski@uj.edu.pl; Tel.: +48-507-769-829

**Keywords:** SARS-CoV-2, viruses, ionic nanochannels, viroporins, selectivity, Brownian motions, ultrasound, numerical simulations

## Abstract

The ionic E-nanochannel (viroporin) is the weak point of SARS-CoV-2, the virus responsible for the (still threatening) COVID-19 since it is vital to the virus’s budding and propagation. Therefore, targeting it to disable its functions ought to incapacitate, or at least weaken, the virus. The ionic currents inside this channel could be affected and disturbed by direct physical attack via the actions of external fields. The paper presents the first step towards the application of such methods in the fight against the current pandemic, numerical simulations of external fields’ impact on ionic currents through viral channels. These simulations—based on the actual, detailed physical nanostructure of ionic channels, measured experimentally and reported in the literature—show that external physical fields can diminish the channel’s currents and that the lower the channel’s selectivity, the stronger the effect. Simulations suggest that SARS-CoV-2 E-viroporin is almost non-selective, which means that the whole virus ought to be highly vulnerable to the actions of external physical fields, much more vulnerable than the much more selective human cell ionic channels. If corroborated by experiment, this observation may result in an innovative method of dealing with the recent pandemic caused by SARS-CoV-2 and other similar viruses.

## 1. Introduction

All existing and ongoing research related to combatting the present COVID-19 pandemic is focused on chemical [1], biological, and social measures (drugs, vaccines, isolation of infected). Among the numerous proposals for battling the source of this pandemic, SARS-CoV-2, the use of *physical* means against viruses is largely neglected. There is one well-documented recent suggestion to use electromagnetic (microwave) radiation for the denaturation of spike proteins [2]; however, cursory mentions of the possible use of electric, magnetic, and electromagnetic fields lack precise data or even specific suggestions.

Meanwhile, recent literature about the properties of SARS-CoV-2 and other coronaviruses [1,3,4,5,6,7,8], together with a small number of earlier papers [9,10,11], point out that the ionic channel (a.k.a. viroporin) E—or rather, strictly speaking, the flow of ions through the viroporin—plays a substantial role both in the budding and propagation of the virus and in its virulence. This implies that the weak point of SARS-CoV-2 should be looked for in exactly that nanochannel and suggests that every action leading to viroporin’s damage should be useful in the fight against the pandemic. Still, even though the physical nanostructure of this virus’s E-channel has recently become known in great detail [3], no publications currently use this research.

In order to start filling these gaps in scholarship, this paper proposes to consider the hypothesis that the activity of viroporin E, the junction between the virus and the cytoplasm of the infected cell, which is responsible, among others, for the replication of the virus, and can be disturbed directly by the action of external physical fields. Of these, the most promising ones appear to be ultrasonic and magnetic, which are deemed safe for humans. Fundamental physics implies that these fields do act on the ions’ motions and, therefore, will influence the ionic nanochannels’ currents. Still, the strength of that influence cannot be estimated for the lack of experimental data. Available means used in the fight against COVID-19 (drugs, vaccines) target specific fragments of the virus’s biological structure and therefore are affected by the fast changes (i.e., mutations) of this structure. Physical forces are not directed at the virus itself but at the motions of ions (currents) within the virus’s weak point, i.e., its ionic channels. Thus they are much less sensitive to these mutations, which makes them a universal instrument in the struggle against viruses. Moreover, tunnelling nanotubes (one of the routes for SARS-CoV-2 spreading), described recently in [12], should be vulnerable to at least one of the physical fields, i.e., ultrasound. The proposed hypothesis can be verified experimentally, either by measurements in vivo, on infected organisms, or in vitro, by measuring ionic currents through the E-channels synthesised in so-called black films (artificial simplified cell membranes) from the respective E-proteins, using the techniques described, e.g., in [10,11]. Yet another possibility for basic verification of our hypothesis is the simulation of the aforementioned ionic currents. They ought to be based on a sufficiently good model of the ion motions inside the channel and use a reliable model of the ionic channel and reliable experimental data. This would provide guidelines for further experiments on active viroporins. Such simulations are presented in this paper.

The aim of the research presented below is, therefore, twofold: (i) To build a realistic model of an ionic nanochannel, (ii) Assess to what extent the external ‘attacks’ immobilise the channel, or at least sufficiently decrease its activity, which in turn will diminish the virulence and reproductivity of the virus. A ‘value-added’ of this method, if corroborated experimentally, would be that external fields act immediately during the application, while the response to drugs requires time, and, as pointed out above, the former is less sensitive to the viruses’ mutations.

The present paper discusses the construction and application of such a model. To this end, detailed information about the physical structure of the ionic biological nanochannels is necessary.

## 2. Results and Discussion

### 2.1. Structure of the Channel

**Figure 1:**
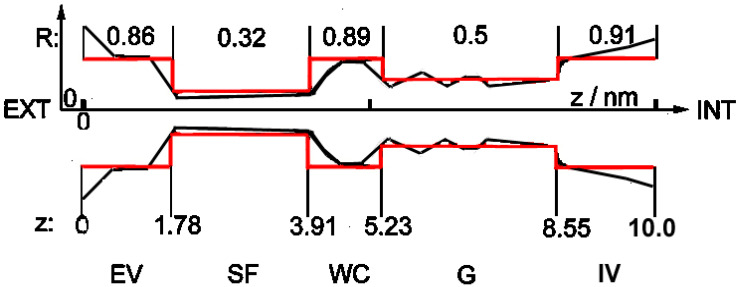
The general geometrical structure of a biological ionic nanochannel. Red lines: schematic presentation of the simplified structure of the channel in question; black shape: a sketch of the geometrical structure of ionic biochannels based on KcsA K^+^ channel presented in Figure 1 in [13] and Figure 3 in [14]. The similar general structure of the SARS-CoV-2 E-viroporin is discussed in [3]. EV: external vestibule, SF: selectivity filter (single-file motion), WC: water cavity, G: gate, IV: internal vestibule. R: [numbers, nm] describe radii *r* of cylindrical fragments of the simplified model, z: [numbers, nm] describe distance along the channel longitudinal axis from the external entrance (z = 0) towards the interior (z = 10).

Until recently, not even the general physical structure of viral E-channels was known. This problem was solved at the end of 2020 with the publication in which the atomic structure of SARS-CoV-2 viroporin, together with the details of its critical part, was published [3], clearly showing that it is almost identical to the known structure of the bacterial potassium channel KcsA [13,14] (cf. Figure 1, heavy black shape), and of practically all currently known structures of ionic channels, both bacterial and eukaryotic [15,16,17]. One of the most important properties of this structure is its strong asymmetry along the longitudinal axis, together with relatively good axial symmetry. The *detailed* structure of the selectivity filter section is also known (cf. Figure 2 in [13] and Figure 9 in [3]).

This result can serve as the ultimate proof that practically all known ionic biochannels, including viroporins, have the same *gross geometrical* structure shown schematically in Figure 1.

Nevertheless, detailed structures of, and roles played by the remaining large parts of the channel, shown in Figure 1, have not been published. Even the maximal number of ions present simultaneously in the channel is unknown. It is assumed that only three ions inside the selectivity filter block the channel [9,15,16,17].

It is the electrostatic structure of the channel that is more important for calculating ionic currents flowing through a channel; of particular consequence are localised charges inside the channel’s walls. The latter can be computed from the measured structure of proteins from which the channel is built. However, the structure will describe only *an empty channel*, while the presence of charged ions inside the channel induces new changes in its structure (cf. [14]) (cf. Methods, below).

The very strong similarity of all known *gross geometrical* structures of ionic biochannels does not mean, however, that they have the same *detailed electrostatic* structure. Earlier results presented in [7,10,11] imply that several specific properties of viral nanochannels differ significantly from bacterial and eukaryotic ones. The most important difference, and probably the most dangerous from the medical point of view, is the substantially lowered selectivity of viroporins compared to non-viral channels, which are very strongly anion–cation-selective (cf., e.g., [15,16]). In particular, simulations in [3] point out that the E-channel of SARS-CoV-2 is very strongly non-selective and allows for the passage of Cl^−^ ions together with Na^+^ and K^+^, with only a residual selectivity between cations and anions (K^+^ and Na^+^ vs. Cl^−^ is about 3-5:1). According to [3] (the conclusion from their model and analysis of the electrostatic field inside the SARS-CoV-2 E-channel), this means that there is (almost?) no charge in the selectivity filter part of the channel. Such residual selectivity implies, in turn, that other charges, somewhere inside the channel, should be of consequence. It is noteworthy that the previous version of this particular virus, SARS-CoV-1, the source of the 2002 epidemic (in earlier literature simply SARS-CoV), is much more selective (about 90:1). Meanwhile, the sequences of amino acids in the E-proteins of both these viruses differ only in a few places, as is shown by data published in [3]. 

### 2.2. The Model

The motion of an ion is described in our model as anomalous Brownian motion in a very confined space [18,19] and with reset [20,21]. This means that an ion moves back and forth in an irregular manner, changing its position **x**(t) to **x**(*t + δt*) within the channel every microtime step *δt* (in simulations usually 10^−9^–10^−7^ s), where the vector **x** denotes the position within the channel described in Figure 1 as (*R,z*). Moreover, (i) An ion can enter/leave the channel’s confined space only through one of the vestibules; (ii) It is assumed that reset means that an ion can enter the channel from outside only when another has just left the channel. Ions move due to actions of several natural, external and internal force fields (built-in and induced ones, electrostatic, chemical, friction, and stochastic; the latter is known as ‘noise’), as well as under the influence of the aforementioned disturbing external physical fields. The registered ionic currents (in pA) passing through the channel are calculated as the numbers of ions which enter the channel by one of vestibules and leave it through the other one during the macrotime step Δ*t* = 0.0001 s, the latter being the recent standard time window of measuring devices.

Performed simulations correctly reproduce all so-called observables previously known in the literature, i.e., subtle physical characteristics coded in the measured series of the channels’ ionic currents, including such abstract ones as non-Markovianity, ergodicity in the wide sense, α-stable distributions of series of subsequent open and closed channel states, etc. This suggests that the model, together with equations of motion, correctly describes the internal dynamics of the ion behaviour.

Several additions to the scheme presented above were examined; among these ageing [22] of the channel seems to be the most promising. Results of simulations indicate that all variants give roughly the same results concerning the primary objective of the present research, i.e., how to dampen ionic transport through the channel. Therefore, following [23] and for the sake of clarity, we shall consider a *simplified version* of the model:

(i) The channel in this simplified shape is built of subsequent cylindrical segments with different radii (red structure in Figure 1). Cylindrical symmetry of the segments is assumed, but as noted above, the whole shape of the channel is strongly asymmetrical along the *z*-axis.
(ii) The trajectory of only one ion at a time is analysed in detail; others remain in the background as charges.
(iii) Proper Open/Closed (high/low current) sequences of the channel’s state are programmed based on empirical distributions (and other signatures) of the eukaryotic Kv channel [24], as determined in [25].
(iv) According to the observation in [3] that the SARS-CoV-2 E-channel has a very low charge in the selectivity filter region, and that there is some negative charge somewhere inside the channel (cf. above: Introduction), one constant charge inside the channel was introduced, located near the entrance from the external vestibule to the narrow internal parts. Three types of channels were examined: low-selectivity (ca. 3–5:1) *viral* with charge −0.01 e; medium-selectivity (ca. 90:1) *viral* with charge −2.5 e; and high-selectivity (above 1000:1) pro- and eukaryotic with charge −5.6 e, anion and cation charges being −1 e and +1 e, respectively.

Simulations show that corrections due to the omitted details do not affect the main conclusions described below.

### 2.3. Simulations of Influence of External Physical Fields

Acoustic (ultrasound) and magnetic fields were chosen for these simulations as the most promising physical actions in the context discussed; they have a long history of being used as diagnostic tools safe for humans, and they easily penetrate the whole body, therefore can be used to attack active viruses inside host cells. Ultrasound acts as ‘kicks’ of a frequency of about 10^−7^–10^−6^ s applied to the channel. This results in changes in the ion current, either due to disturbances in the channel’s shape or the ion’s trajectory through the channel. Magnetic field, when applied from without, can act continuously on an ion through the so-called Lorentz effect (cyclotron motion [26]), which will throw the ion trajectory from the easiest, low-friction path towards the channel walls with a much higher friction, thus decreasing the effective current. In both cases, the dampened current results from randomised changes in friction.

Due to the differences in actions of these fields, the application of combined fields with different chosen strengths and frequencies promises the best results. Additional value of the application of ultrasound lies in the following: it is well-known that *imageing* by ultrasono*graphy* does not work in the lungs because of the scattering of sound waves by small air bubbles present in the tissue. However, for the same reason, chaotic reflections of ultrasound *waves* on the air–tissue phase boundaries should enhance the amount of energy directed against viroporins. Moreover, the ultrasonic field could be used simultaneously with chemical methods in order to enhance the action of appropriately applied antiviral drugs directly on the ‘live’ virus inside the infected cell. A method recently in development (*in statu nascendi*) called sonoporation [27] appears promising for such therapy. Actually, a mixed strategy should be optimal; ultrasound strengthened by a magnetic field, with simultaneous administration of drugs directly to the lungs.

**Figure 2:**
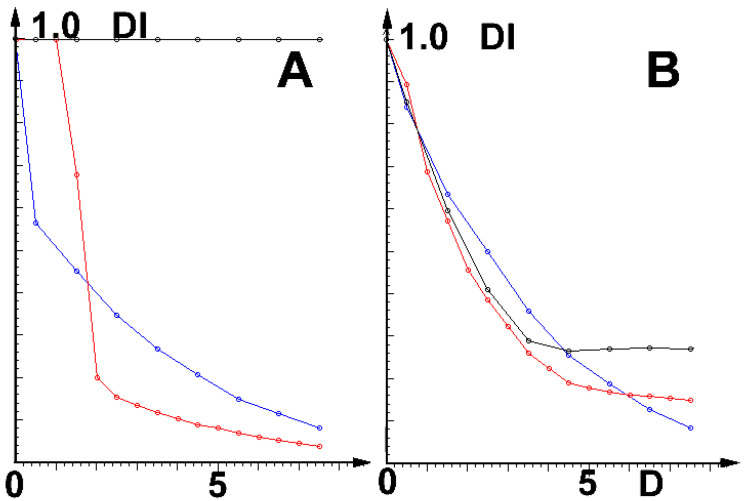
Relative diminishing of current *DI* = < *I_D_*(*t*)/I_0_(*t*)> as the function of field strength *D* (in arbitrary units). (**A**): Cation, (**B**): Anion currents; black: prokaryotic and eukaryotic (incl. human), red: viral (incl. SARS-CoV-1), blue: SARS-CoV-2 channels.

The main results of the simulations are shown in Figure 2:

Note that at low field intensities (*D*), the channel of SARS-CoV-2 is much more vulnerable than its earlier version, SARS-CoV-1. In stronger fields, the reactions of both viruses are roughly the same.

Parameters (including *D* and values of effective channel charges near the entrance—cf. Table 1) are chosen to keep cation currents near 20 pA (typical measured experimental ranges) and the assumed selectivity strength in the presumed values. Similar results were obtained for the ultrasound field. The question remains whether the effects discussed here will be sufficiently strong to be medically relevant.

### 2.4. Results

The main results of presented research are as follows: performed simulations, based on the actual, experimentally measured and reported in the literature, detailed physical nanostructure of ionic channels, show that the actions (shocks, cyclotron effect) of external physical fields can diminish the channel currents.

Most importantly, the less selective the channel, the stronger its reaction to disturbances; the effect is strongest on the very low-selectivity (cation:anion ca. 3–5:1) channel E of SARS-CoV-2 and a few other coronaviruses [11]). Moreover, at low field intensities (cf. Figure 2), this virus is much more vulnerable than the cause of the earlier local epidemic, the medium-selective SARS-CoV-1 (cation:anion ca. 90:1). Of even more importance is the result that E- viroporins of both these viruses are much more vulnerable than the human cells’ channels (cation:anion ca. 1000:1). Because of the direct relationship between the activity of E-viroporins and their budding and replication (cf. Introduction), this means, in turn, that the relationship between viral propagation and channel selectivity exists. The simulations also show that selectivity depends on the internal charge in the channel’s walls near the selectivity filter. This result is in agreement with the earlier observation [3] that SARS-CoV-2 E-viroporin has almost no charge in that region. Moreover, the results shown in Figure 2 imply that very strong ultrasonic and magnetic fields should be safe for use with infected humans (and mice, etc.).

The results described above, together with earlier observations in the literature, suggest the following treatment method in the SARS-CoV-2 pandemic: as remarked above, due to the differences in the actions of ultrasound and magnetic fields, the application of their combined actions with different chosen strengths and frequencies promises the best results. Moreover, ultrasound should enhance the action of appropriately applied antiviral drugs directly on the ‘live’ virus inside the infected cell by the aforementioned sonoporation [27]. When heads (*transducer probes*) of existing imaging USG devices are used as generators of ultrasonic fields, the simultaneous actions of several such devices with different frequencies are recommended.

### 2.5. Discussion

To sum up, a mixed strategy should be optimal; ultrasound strengthened by a magnetic field, with the simultaneous direct application of drugs to the lungs. The additional bonuses of this method: as observed in the introduction, are: (i) It should be much less vulnerable to the continuing mutations of the virus; (ii) Tunnelling nanotubes (a route for spreading SARS-CoV-2) should be vulnerable to ultrasound, as described recently in [12].

Other results: It seems worth noting that the strong asymmetry of the shape of the channel, and related asymmetry of the internal electrostatic fields, lead to several interesting physical effects observed earlier in synthetic channels [28,29,30]. In particular, such asymmetry can be used for pumping the ions against the concentration gradients [31,32]. Our initial simulations of the channel shown in Figure 1 indicate that biochannels can also be converted into ionic pumps, e.g., under patch clamp with an oscillating holding potential. Another similar effect is the so-called asymmetric nano-diffusion [33,34,35] (in particular, cf. sect. 5 in [35]).

## 3. Methods

### 3.1. Ionic Biochannels

The physical parameters of ion channels were: length of about 10 nm, internal diameter of a few nm, in the narrowest part (selectivity filter—cf. Figure 1) radius of less than 1 nm, strong asymmetry along the *z*-axis. Inside the channel, ions move under very strong external electrostatic fields; a typical potential difference along the channel, i.e., between the outside and inside parts of the cell membrane (a few nm thick), in which the channel is immersed, is about 50–80 mV, i.e., it exceeds ten MV/m; during measurements. The typical external potential difference along the axis of the channel is again tens of mV, i.e., a few MV/m. In the narrowest part of the channel (selectivity filter), ions must be stripped of their water shells and go in single-file formation. All this means that the motion of an ion inside a nanochannel is very far from the standard (macroscopic) continuous diffusion.

#### 3.1.1. Basic Properties

Ionic channel currents *I(t)* (i) are stationary in a wide sense; (ii) They consist of irregular sequences with non-Gaussian distributions of high (open state, O) and low (closed state, C) values of *I(t)*; (iii) Short fragments of *I(t)*, either open or closed, are strongly irregular with characteristics of anomalous Brownian motion with non-Gaussian distributions [24]; (iv) Are non-Markovian [25,36,37,38] (there are no such data for viroporins); (v) Power spectra *S*(*f*) have characteristics of flicker (‘pink’) noise, i.e., *S*(*f*)~(1/*f*)^α^ with *α* near 1 [38,39] (no such data for viroporins); (vi) Distribution of *I*(*t*) is bistable, results are well-approximated by superposition of two long-tailed asymmetric stable distributions, dwell-time distributions fit well to stretched exponential functions, different for open and closed fragments [24] (there are no such data for viroporins).

#### 3.1.2. Comments

Ad (i): Otherwise, the whole organism would not function properly.

Ad (ii): Open/Closed sequences appear in all known biochannels, including viroporins (cf. Figure 4 in [10], Figure 6 in [4], Figure 1 in [5]), and in some ‘soft’ synthetic channels [38]. However, the mechanism and role of these sequences are not sufficiently understood. A possible explanation of this seemingly uneconomic behaviour can be given by the ageing [22] of the channel, i.e., the time-dependent changes of free parameters of the channel’s internal structure, due to the interaction with passing ions. Ageing occurs in the channel’s open state (high conductivity); during a subsequent period of low conductivity (closed state), the structure recovers slowly and the channel returns to its initial state.

Ad (iii): This does not contradict the stationarity in the wide sense; the use of a local-scale Lévy process assures that the two important properties are satisfied; *stationarity in wide sense* of the channel’s current (scale 0.0001 s) and taking the local time flow (scale ps) into account. This also can include the ageing of the channel.

Ad (iv): The non-Markovianity of a given stochastic series indicates the presence of a specific type of temporal correlation, frequently called ‘memory of the past’ [19,36].

Ad (v): The mechanism of the appearance of (1/*f*)*^α^* (‘flicker’, ‘pink’) noise is unknown. According to Bezrukov’s conjecture [40], flicker noise in bio-nanochannels is caused by “the equilibrium conductance fluctuations related to the conformational flexibility of the channel pore constituents”; in turn, according to [39], fluctuations of conductivity are the noise’s source. Both these statements can be reconciled by assuming that partially and randomly mobile small compartments of the inner channel surface cause random fluctuations of local conductivity. Flicker noise with α = 1.1 ± 0.1 is found both in soft biological Kv and synthetic PET channels (‘dangling ends’ within the latter), but not in synthetic Kapton ones (smooth inside surface) [38], which apparently supports the conjecture cited above. It is noteworthy that ‘compartments’ and ‘dangling ends’ are clearly visible in the selectivity filter structures of the KcsA channel [13] and the SARS-Cov-2 E-channel [3], Figure 9. These results are important because, together with stationarity in the wide sense of channel currents, they imply, by virtue of the Wiener–Khinchin theorem, that *I(t-s)*~|*t-s*|^−α^ asymptotically, and by virtue of Khinchin’s theorem, series {*I(t)*} are asymptotically ergodic. However, short sequences of *I(t)* are usually non-ergodic, as our simulations show.

Ad (vi): The statistical properties of the long-measured series *I(t)* are known for the potassium Kv eukaryotic channel [25] and are described in [24].

### 3.2. Details of the Model

As stated above, the motion of an ion is described as anomalous Brownian motion in a very confined space [18,19] and with reset [20,21], under several internal and external fields of force:

(i) Electrostatic interactions of ions with remaining ions, and with both static and induced charges within the channel itself;
(ii) The differences in the concentration of given ions inside and outside the cell membrane (‘chemical’ force);
(iii) Friction force: data from [3,13,14], related to the atomistic structures of the KcsA channel and E-viroporin of SARS-CoV-2, suggest that the inner surfaces of the channels are coarse, both in a mechanical and electrostatic sense;
(iv) Stochastic forces (noise): always present thermal noise (Gaussian White Noise) and more or less random influences of neighbour processes [18,19]; of note, the channel itself moves randomly in two dimensions inside the semi-fluid cell membrane [41]. This means that the Brownian motion of an ion is no longer normal diffusion (Wiener process) *W(t)* in external fields, but an indefinite anomalous one.

The model of anomalous Brownian motion is used in the non-simplified model of the Lévy process [19] *L_H_*(τ) with the Hurst exponent *H* treated as a free parameter, with *τ* measuring the running time from the entrance of the tracked ion into the channel to its exit. The Lévy process means the convolution of white noise (or another process) with running time, which introduces a kind of local memory (i.e., the non-Markovianity of the process). This takes the local time flow of the motion of a single ion (scale 10^−6^–10^−9^ s) into consideration during its passage inside a channel, and at the same time, assures the stationarity in a wide sense of the whole channel’s current (scale pA, measuring window 10^−4^ s), necessary for correct physiological functioning of the channel. In the simplified model, the Lévy process is substituted by a scaled Wiener process, which leads to the same conclusions, but is simpler. Both versions enable the introduction of corrections due to the ageing of the channel.

The set of free parameters was chosen so that the number of ions passing through the channel during Δ*t* = 0.0001 s (experimental window) is about 25 pA. This is the typical range of experimental data, including the Kv channel [25], viroporins [9], and synthetic channels [28,29,30]. For each series of simulations, 100–300 different realisations of the thermal noise were computed to obtain appropriate average values. At the output, the number of ions (charges) leaving the channel during the time interval Δ*t* = 0.0001 s is rescaled to pA.

### 3.3. Equations of Motion

Let *i,j* denote numbers of ions, *k*—any element of the channel: ion *i*, charge of specific place within walls, etc., and *x_i_* denote either *z*- or *r*-coordinate of *i*-th ion. Then equations of motion of ions can be written in the discrete form of anomalous Brownian motion:

*x_i_*(*t*) = *x*_*i*0_ + *W_x_*[*A_x_ ξ*(*t*) − *ρ*(*t*) + *V*_0_ + Δ*c* + ∑*_k_*
*ϕ_i,k_* + ∑*_j_* bar_*j*_ (1) ]

*ϕ_i,k_* = *F*_0_
*q_i_ q_k_*/*L_i,k_*^2^,  *L_i,k_*^2^ = |*z_i_*^2^_+_
*r_i_*^2^| − |*z_k_*^2^_+_
*r_k_*^2^|, (2)

*ρ*(*t*) = *ρ_c_* (*t*) + *ρ_m_* (*t*) + *ρ*_ext_, (3)

*ρ_c_* (*t*) = *ρ*_0_
*e_i_ e_k_* [*y*_*i*0_/(1 − *y*_*i*0_)]^2^,   *y_i_* = *r_i_* (*z_i_*)/*R_i_*(*z_i_*) (4)

bar *_j_* = *e_i_ e*_bar*j*_/(d*z_j_*^2^ + 10.0^−6^), d*z_j_* = *z*_bar*j*_ − *z_i_*. (5)

where *W_x_*, *A_x_*, Δ*_C_*, *F_0_*, ρ_0_—free parameters, for which there are no observational data, and which, moreover, scale subsequent terms in the nanometer dimension. *R(z_i_)*—radius of the channel at *z_i_*, *x*_i0_ = *x*_i_ (*t* − *δt*), *δt*—micro time-step (usually 1–100 ns), ρ—effective friction, being the sum of electrostatic, mechanical, and external ones; ρ*_m_*—the deceleration by sticking-out peptide fragments (the latter being an analogy of ‘dangling ends’ in synthetic channels [37] cf. respective figures. in [3,13,14]), ρ*_ext_*—any additional external disturbances, bar—electrostatic interactions of ion *i* with both static and induced charges within channel’s walls.

The last point needs a more detailed explanation: the ion entering or passing through the channel ‘feels’ the ‘barrier’ of electrostatic force from the effective charge(s) of the channel’s walls, the latter being the sum of permanent structural charges present and the charges induced there by this same ion. The parameters which describe this effect, used in the simplified version of the model for computing the results shown in Figure 2, are presented in Table 1 below.

### 3.4. Conclusions

The methods of dealing with viroporins proposed in this paper need experimental verification. The direct measurements of changes in ionic currents inside host cells containing active viruses are, at present, very difficult, if possible. Measurements by techniques developed in refs. [10,11] (synthesised E-viroporins from viral E-proteins immersed in planar lipid bilayers) are also difficult and expensive and yield not entirely reliable results: the measured in this way currents are averages of currents flowing through several viroporins, instead of being measured by a patch clamp technique in a single channel; channels are ‘naked’, i.e., deprived of their natural protein cover (note that, according to [42], the behaviour of processes inside confined dielectrics depends on the surroundings of the investigated system, i.e., in our case, of the channel itself).

The most definitive verification of the presented hypothesis will be a direct experiment in vivo, which ought to be possible (or even easy?) now, due to the appearance of a new strain of mice designed to be models of SARS-COV-2 [43].

If the hypothesis is verified by experiment, it would be recommended to perform measurements on other types of viruses which still have the potential of threatening humanity, such as pox, Ebola and mosquito-transferred viral diseases, in order to establish their selectivity and vulnerability to the proposed method.

## Figures and Tables

**Table 1:** Barrier parameters.

	SARS-CoV-2	SARS-CoV-1 et al.	KcsA, Kv, et al.
chbar_10_	−0.01	−2.5	−5.6
∧_c_	−2.1	−1.1	−1.1
∧_a_	0.5	0.32	0.01
z_bar1_	4.0	2.5	1.77

Parameters—parameters used in the program when computing the results shown in Figure 2; barrier (‘bar’ in the program = force from internal charges inside the channel, both native in the channel’s walls (chbar_10_) and induced by the entering ion (ch_ion_*∧); ∧—induction parameter; bar = ch_ion_*chbar_1_/(dz_1_*dz_1_ + 10^−6^); dz_1_ = (zbar_1_ − z_1_); z_1_: ion position along the channel’s *z*-axis; ch_ion_: charge of the entering ion; if(ch_ion_ > 0) ∧ = ∧_c_ (cation) else ∧ = ∧_a_ (anion); chbar_1_ = chbar_10_ − ch_ion_*∧.

## Data Availability

All data are taken from cited references. The source code of the computer program was written by the present author and can be obtained on reasonable demand.
